# Connecting Families to Their Health Record and Care Team: The Use, Utility, and Impact of a Client/Family Health Portal at a Children’s Rehabilitation Hospital

**DOI:** 10.2196/jmir.6811

**Published:** 2017-04-06

**Authors:** Gillian King, Joanne Maxwell, Amir Karmali, Simon Hagens, Madhu Pinto, Laura Williams, Keith Adamson

**Affiliations:** ^1^ Bloorview Research Institute Holland Bloorview Kids Rehabilitation Hospital Toronto, ON Canada; ^2^ Department of Occupational Science and Occupational Therapy University of Toronto Toronto, ON Canada; ^3^ Holland Bloorview Kids Rehabilitation Hospital Toronto, ON Canada; ^4^ Canada Health Infoway Toronto, ON Canada; ^5^ Faculty of Social Work University of Toronto Toronto, ON Canada

**Keywords:** disability, engagement, health information, caregivers, children with disabilities, patient portals, electronic health records

## Abstract

**Background:**

Health care portals have the potential to provide consumers with timely, transparent access to health care information and engage them in the care process.

**Objective:**

The objective was to examine the use, utility, and impact on engagement in care and caregiver-provider communication of a client/family portal providing access to electronic health records (EHRs) and secure, 2-way e-messaging with care providers.

**Methods:**

We conducted a prospective, mixed-methods study involving collection of caregivers’ portal usage information over a 14-month period (from portal introduction in January 2015 to the end of the study period in March 2016), a Web-based survey for caregivers administered after a minimum of 2 months’ exposure to the portal and repeated 2 months later, and focus groups or individual interviews held with caregivers and service providers at the same points in time. The survey assessed caregivers’ perceptions of the utility of and satisfaction with the EHR and e-messaging, and the portal’s impact on client engagement and perceptions of caregiver-provider communication. A total of 18 caregivers (parents) completed surveys and 6 also took part in focus groups or interviews. In addition, 5 service providers from different disciplines took part in focus groups or interviews.

**Results:**

Although usage patterns varied, the typical pattern was a steady level of use (2.5 times a month over an average of 9 months), which is higher than typically reported use. The portal pages most frequently accessed were the home page, health record main page, appointment main page, and reports main page. The Web-based survey captured caregivers’ perceptions of usefulness of and satisfaction with the EHR and portal messaging, as well as the portal’s impact on their engagement in care and perceptions of caregiver-provider communication. The surveys indicated a moderate degree of utility of and satisfaction with the portal features, and a low but emerging impact on engagement in care and caregiver-provider communication (survey scales measuring these outcomes displayed excellent internal consistency, with Cronbach alpha ranging from .89 to .95). Qualitative themes from focus groups and interviews supported and extended the survey findings. Caregivers and service providers saw appreciable information benefits and provided recommendations to increase portal use and utility. Caregivers focused on the scope of organizational adoption of the portal system and indicated their hopes for the future of the portal, whereas service providers were concerned about how to best manage their investment of time and effort in preparing client-friendly reports and messaging clients via the portal.

**Conclusions:**

Overall, the findings show the promise of the portal and the need for ongoing evaluation to show the portal’s ultimate potential in enhancing engagement in care and communication with care providers.

## Introduction

Health care portals have the potential to improve consumers’ access to information, engagement in care, and health outcomes [1]. Few children’s hospitals in North America offer online portals to families [2] and, to our knowledge, there are no published research studies of portal use, utility, or impact in pediatric rehabilitation hospitals. Through electronic access to health records and e-messaging (secure 2-way messaging), clients and families may feel a greater personal connection to care and partnership in the care process.

In pediatric rehabilitation, the chronic nature of children’s difficulties and the need for parental support have resulted in widespread adoption of family-centered care, which stresses the importance of engaging in partnerships with care providers and exchanging information [3]. Engagement in care refers to a sense of meaningful involvement, true collaboration, or authentic participation [4], and the active involvement and full investment of children and parents in therapy is presumed to be essential to achieving positive client outcomes [5,6]. Similarly, good client-provider communication is considered essential in the delivery of children’s rehabilitation services [7]. Effective communication is strongly associated with client satisfaction [8,9], and studies have indicated that communication plays an important role in the ongoing client-practitioner relationship, and especially in engaging clients [10].

### The Promise of Health Care Portals

Consumers are asking for the ability to view their appointment schedules online, have electronic access to documentation and other resources, and have their questions answered over the Internet. E-scheduling, e-visits, and e-messaging are seen as important portal functions [11], as they allow information exchange and can potentially enhance consumers’ ability to manage their own health and health care [1]. By having access to a portal that does more than just provide information and data, consumers can become more active participants in their own health care [12]. Thus, portals have the potential to provide more personalized, collaborative, and effective care. There is, however, limited (but growing) research information on the use of electronic communication in health care, including electronic health records (EHRs) [1,13].

Research on EHRs has typically focused on adult medical care and management of chronic diseases such as rheumatoid arthritis [14] and diabetes [15] rather than children’s chronic conditions [16]. We searched for articles on portals in pediatric rehabilitation hospitals (using “pediatric” or “paediatric” with “online” and “portal,” with searches limited to 2015 and more recent). We found articles on portals for children with mental health conditions [17], with asthma [18], and those receiving acute care in a pediatric hospital [19]. We found no articles on portals in pediatric rehabilitation hospitals. A recent evaluation of a portal at a Canadian children’s acute care hospital indicated that, at 3 months after EHR implementation, service providers perceived a negative impact on their efficiency, productivity, and the quality of care they provided, whereas patients and families did not perceive a negative impact on care [20].

Even for adult chronic disease management, there is sparse evidence to support the ability of personal health records and EHRs to improve efficiencies, reduce costs, and improve outcomes [15,21]. Most existing research on health care portals has examined the frequency of use of their features, satisfaction with the portal, or efficiencies in health care utilization (eg, number of office and emergency department visits, phone calls) [22-24].

Systematic reviews on the effects of portals with EHR access [25-27] indicate that few studies have investigated outcomes concerning patient engagement or empowerment, with studies typically providing nonsignificant findings. Kruse et al [27] found only 27 studies of relevance to impact—those measuring meaningful outcomes such as patient participation in medical decisions, patient-provider communication, or satisfaction. For example, a study by van der Vaart and colleagues [14] found that approximately 44% of patients with rheumatoid arthritis reported feeling more involved in treatment as a result of access to their EHRs; however, significant pre-post differences in empowerment were not found. Thus, research is needed to substantiate “meaningful use” impacts [1,15].

### Summary of Research Gaps

To our knowledge, there has been no research on client portals for pediatric rehabilitation service users. As well, the literature has focused on portal use, satisfaction, and service utilization outcomes, and there is a recognized need to examine meaningful outcomes such as client engagement in care and communication with providers. These 2 outcomes are of fundamental importance in pediatric rehabilitation, as well as in medical services for people with chronic conditions [27]. Furthermore, although some studies have tracked portal use over time (eg, [28]), studies have not examined portal impact prospectively. Examining portal use, utility, and impact over time provides an opportunity to see how users access portal features and may also indicate emergent short-term impacts [29], such as enhanced engagement in care. Since studies typically evaluate portal features in isolation [27], there is benefit in examining client perceptions of the introduction of a full portal over a period of time. Lastly, the literature indicates the value of mixed-methods studies, which are rare in portal research [27].

### The Client Portal at Holland Bloorview Kids Rehabilitation Hospital

Holland Bloorview Kids Rehabilitation Hospital (Holland Bloorview) in Toronto, Ontario, is Canada’s largest children’s rehabilitation hospital. The hospital is a provincial resource for children with cerebral palsy, acquired brain injury, muscular dystrophy, amputation, epilepsy, spina bifida, arthritis, cleft lip and palate, autism, and other physical and developmental disabilities. Holland Bloorview’s vision is to create a world of possibility for kids with disability by embracing client- and family-centered care, and participating in applied research and education.

In early 2015, Holland Bloorview launched a consumer portal with the ultimate goal of helping clients and their caregivers (family members, most typically parents) take an active role in managing their own or their children’s care. The consumer health portal (called connect2care) was developed in partnership with clients and families [2]. Connect2care provides clients and families with electronic access to their medical records, online appointment cancelling and booking features, transparent and timely access to clinical documentation, and e-messaging to connect with their care providers.

Beginning in January 2015, enrollment to the portal was initiated, first for clients on the inpatient units, and then also for ambulatory and community programs. In this first phase, the functions available included the ability to view the client’s schedule and visit history, view and print clinical notes, and update demographic details. In May 2015, improvements were made to the viewing of laboratory and microbiology test results, and new processes were established to increase the number of clinical notes that would flow to the portal (allowing providers from additional health disciplines to share clinical notes). Over the summer of 2015, training of more than 100 health care providers was completed to support this enhanced sharing of clinical notes. In the summer and fall of 2015, messaging functionality was rolled out, allowing secure e-messaging between portal users and their providers. By December 2015, the training of providers for e-messaging was completed.

Enrollment and usage targets for 2015 were established prior to launching the portal, with a target enrollment of 721 users (clients and caregivers), and a target use (unique logins) of 1440. The targets were met well ahead of schedule and, by the end of 2015, there were 869 enrolled users and more than 4800 uses. The adoption rate was approximately 12.41% (869 out of about 7000 unique clients). As well, over 200 staff were live with e-messaging at the end of 2015, including physicians, occupational therapists, physical therapists, speech-language pathologists, social workers, psychologists, therapeutic recreation staff, orthotics and prosthetics staff, ambulatory care nurses, child life workers, and nurse practitioners.

### Study Purpose and Objectives

The purpose of our study was to examine the use, utility, and impact of the connect2care portal from the beginning of portal introduction until the end of data collection 14 months later. To meet this aim, we conducted a prospective, mixed-methods study collecting quantitative survey data and qualitative data from focus groups and interviews with caregivers and service providers at 2 points in time. We adopted a concurrent triangulation approach, in which quantitative and qualitative data were collected at the same time [30] and integrated at the level of interpretation [31]. Our research objectives were (1) to determine caregivers’ portal use over the study period, (2) to examine levels of perceived usefulness of and satisfaction with the portal’s EHR and e-messaging, as well as the portal’s impact on engagement in care and caregiver-provider communication, and (3) to ascertain caregivers’ and service providers’ perceptions of the portal, its utility, and how it could be enhanced.

## Methods

### Study Overview and Design

The study was conducted by a team with diverse organizational roles, including clinical directors, a project manager, researchers, and a family-centered care specialist who is a parent of a client. The team brought different perspectives and backgrounds (social work, occupational therapy, and psychology) to the design of the study and interpretation of the findings.

**Figure 1:**
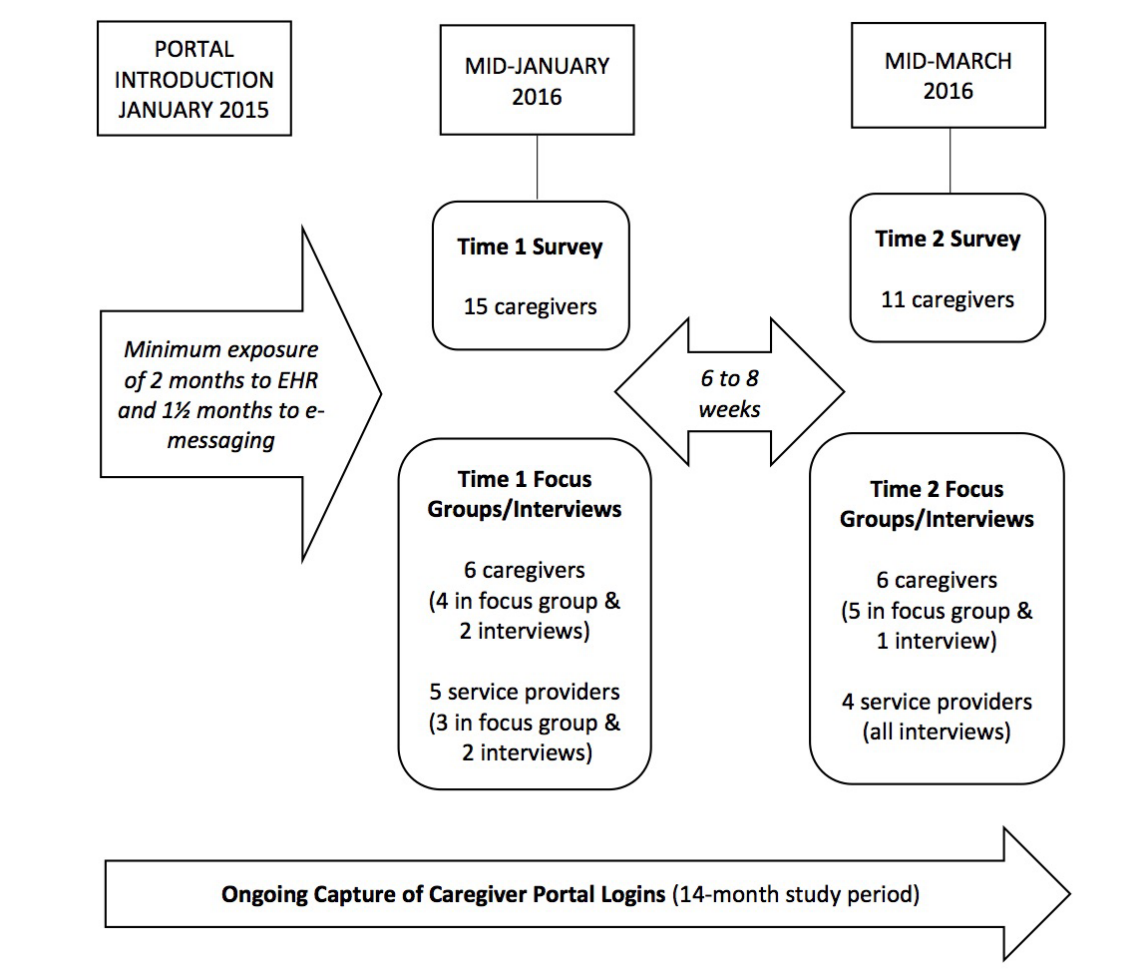
Data collection procedures: logins, surveys, and focus groups or interviews. EHR: electronic health record.

As Figure 1 shows, we used 3 methods of data collection: capture of portal login information, a survey (assessing utility and satisfaction, and impact on client engagement and perceptions of caregiver-provider communication), and focus groups or interviews held with caregivers and service providers. To ensure the opportunity for a base of experience prior to assessment, participants had a minimum of 2 months’ exposure to the EHR and 1.5 months to e-messaging before time 1 assessment. There was an interval of 6 to 8 weeks between time 1 and time 2 measurement points, allowing us to examine the effects of additional exposure to the portal. Caregivers could opt into 1 of 2 arms of the study: survey only; or survey plus focus group or interview. Service providers participated only in focus groups or interviews.

### Participant Recruitment

#### Caregivers

We obtained ethics approval from Holland Bloorview’s Research Ethics Board. Members of Holland Bloorview’s Family Advisory Committee introduced caregivers to the study when they were registering for the portal. Interested caregivers provided their contact information to learn more about the study from a research assistant. Other recruitment methods were (1) study flyers, (2) emails sent to connect2care users, (3) an advertisement posted on the Participate in Research page on the hospital’s website, and (4) messages posted on the hospital’s Facebook page for parents.

We administered study forms for caregivers (invitation, background form, and surveys) online using Research Electronic Data Capture (REDCap), a secure, Web-based app designed to support data capture for research studies [32]; all forms and materials were in English, as this is the language used in connect2care. Other than the ability to read English, there were no inclusion or exclusion criteria. Caregivers (of clients of any age or diagnosis) accessed a link on the portal’s home page that provided access to REDCap. The REDCap link contained a downloadable PDF version of the information letter and a consent form. At this time, caregivers indicated their interest in 1 of 2 study options (survey only or survey plus focus group), and provided their contact information so that study information and consent forms could be sent to them by email. As part of providing consent, caregivers gave permission for their portal usage information to be included in the research.

#### Service Providers

We recruited service providers using an announcement on the hospital’s internal home page, as well as emails with the study information flyer attached. Interested service providers were emailed the background form, which they returned to the research assistant.

### Background Forms

The background form for caregivers captured demographic information (sex, education), and information about their child or adolescent with a disability (primary diagnosis). It also captured Internet use and ratings of Internet skills [14], since lack of Internet experience is the primary barrier to portal use [27]. The background form for service providers captured discipline, education, and years in practice.

### Portal Survey

Since existing surveys did not capture the outcomes of interest, we developed our own. Satisfaction and utility items were informed by surveys developed to evaluate patient access to EHRs (eg, [14,33,34]). Engagement with care items were informed by the Pediatric Rehabilitation Intervention Measure of Engagement for Parents (GK, unpublished measure, 2015), and caregiver-provider communication items were based on constructs from existing surveys [33,35].

We refined the survey items, piloted them with 6 caregivers to ensure the items were acceptable and easily understood, and then reviewed them for health literacy. Caregivers indicated that the surveys took less than 10 minutes to complete. Based on caregivers’ feedback, we clarified the wording (eg, “complete” meant not missing important information), added definitions of key terms to the instruction section (eg, clinical provider team, care, and involved or engaged), and instructed respondents to click on “not applicable” or “to a small extent or not at all” when they had not had relevant opportunities to do what items specified.

The final survey (Portal Survey on Satisfaction and Impact on Care [36]) contained 38 items in 5 sections: (1) utility/satisfaction: client health record, (2) utility/satisfaction: portal messaging, (3) involvement in the care process, (4) impact of portal messaging with service providers (eg, improvements to communication, ability to express concerns and get clarifications, trust or rapport with clinical provider team), and (5) portal overall (ie, satisfaction and utility overall, future intention to use, impact on care).

### Focus Groups and Interviews

Focus groups and interviews were conducted by an interviewer with more than 10 years of interview experience. Individual interviews were conducted (either by phone or in person) when individuals could not attend scheduled focus groups. Focus groups and interviews were audio-recorded and transcribed by a trained transcriptionist, with identifying information removed. Focus groups lasted an average of 48 minutes (range 35-60 minutes), and individual interviews lasted an average of 19 minutes (range 7-34 minutes). Participants were asked to discuss (1) general thoughts about the portal (including satisfaction and the extent to which they accessed the portal, and for what purposes), (2) how the portal could be enhanced or improved, (3) the most helpful or useful parts of the portal, (4) difficulties or issues in using or accessing the portal, (5) needs and expectations regarding the portal, and (6) whether the portal made things more efficient.

### Data Analysis

#### Portal Usage by Caregivers

Decision Support and Health Information Management at the hospital provided the study research assistant with an Excel 97-2003 (Microsoft Corporation) workbook of data covering the 14-month study period. We used this information to create a dataset containing the portal pages accessed by date for each person enrolled in the research. We then used this information to calculate total number of visits and days of use per person, usage across months per person, and the number of times each page was viewed over all participants.

#### Survey Analysis

We analyzed time 1 and time 2 survey data descriptively, given the small sample size. Aggregate scores were calculated for survey scales (the 5 survey sections), and Cronbach alpha was calculated to determine the scales’ internal consistency reliabilities.

#### Qualitative Analysis

We analyzed transcripts using a content analysis approach, which involves coding statements based on key concepts, combining these coded concepts into themes, and then refining the identified themes [37,38]. Team members read all caregiver transcripts. To ensure confidentiality, service provider transcripts were analyzed by 3 team members (not clinical or project directors). Through an iterative process, the lead author then summarized the transcript data into themes, using tables to group and compare related ideas. The credibility and accuracy of data analysis were ensured by using multiple procedures, including maintaining an audit trail [39]. Trustworthiness was enhanced through peer examination and discussion of findings in team meetings, investigator triangulation (various disciplines, perspectives, and roles), and mutual confirmation of the data [40,41].

## Results

### Description of Participants

#### Caregivers

**Figure 2:**
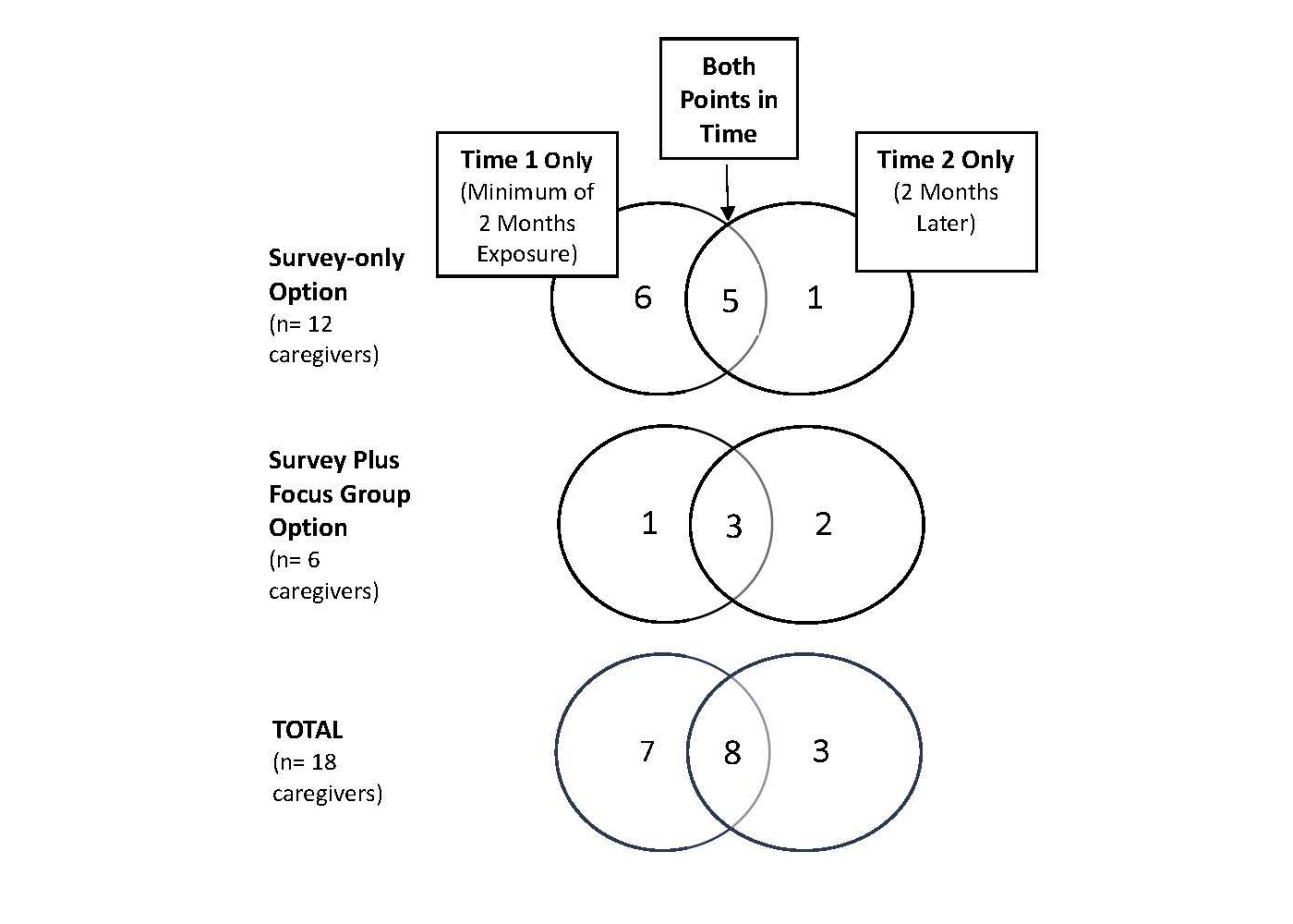
Caregiver participants by study options and 2 points in time.

A total of 18 caregivers took part, some in the survey-only option and some in the survey plus focus group or interview. As Figure 2 shows, some individuals participated at both time points. There were 15 completed surveys at time 1 and 11 at time 2.

Table 1 presents the participants’ characteristics for the entire sample. As well, almost all participants (n=17, 94%) had used the Internet for more than 5 years, and all reported using it daily or several days a week. They rated their Internet skills as either good to very good (n=12, 67%) or average to reasonable (n=6, 33%).

**Table 1:** Caregiver participant characteristics (n=18).

Characteristic		n	%
**Sex**			
	Female	15	83
	Male	3	17
**Age range (years)**			
	20-34	4	22
	35-49	8	44
	50-64	6	33
**Highest education level**			
	High school certificate or diploma	1	6
	College or other nonuniversity certificate or diploma	3	17
	University certificate or diploma (eg, bachelor’s)	9	50
	Postgraduate certificate or diploma (eg, master’s, PhD)	5	28
**Child’s current primary health or development problem**			
	Acquired brain injury	4	22
	Cerebral palsy	4	22
	Autism	3	17
	Spina bifida or hydrocephalus	2	11
	Other (eg, global developmental delay, muscular dystrophy, central nervous system vasculitis or stroke)	5	28

#### Service Provider Participants

Our target was to recruit 8 to 10 service providers for the focus groups, as is typical in focus group research [42]. A total of 5 service providers took part in focus group or interviews: 2 physical therapists, 1 occupational therapist, 1 speech-language pathologist, and 1 nurse; 4 of the 5 participating service providers took part at both time points. They had been in practice 13.5 years on average; 2 had a bachelor’s and 3 had a master’s degree.

### Portal Usage by Caregivers

Table 2 presents portal usage information over the study period, from the introduction of the portal (January 2015) to the end of data collection (March 2016). As Figure 1 shows, we collected login information from first use of the portal, whereas we held surveys and focus groups or interviews only after participants had a minimum of 2 months’ exposure to the portal (to ensure a base of experience). Users joined connect2care at various points over this time period, with an average exposure of 253 days (approximately 9 months). Overall, users logged on to the portal an average of 22.2 times on 19.2 days (2.5 times a month). The most common user access pattern was a combination of home page, health record main page, appointment main page, and reports main page. Thus, users were most interested in their child’s health record, appointments, and reports, as has been reported by others [20].

**Figure 3:**
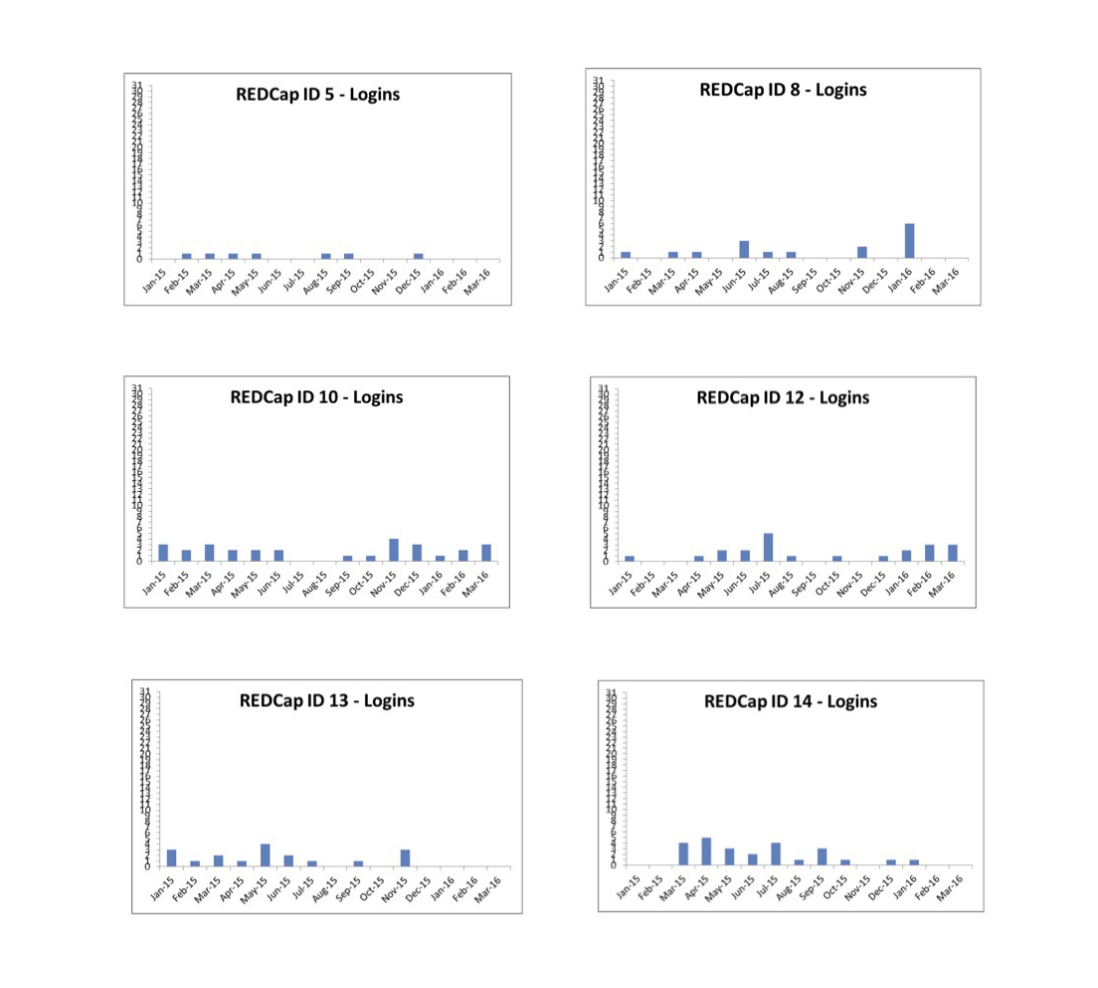
Typical login patterns (total number of logins per month) over the study period for 6 study participants. ID: identification; REDCap: Research Electronic Data Capture.

Login graphs for participants indicated differences in patterns of usage, with some users having high initial use that tapered off, whereas others had no use past their initial portal logon. The typical pattern, though, was a steady level of use (2 or 3 times a month). We chose the graphs in Figure 3 to show typical login patterns over the study period.

**Table 2:** Connect2care portal usage by caregivers over the 14-month study period.

Variable	Mean	Range
Number of days with exposure to connect2care (last session date minus first session date)	253	1-433
Number of times logged in to connect2care	22.2	1-87
Number of days logged in to connect2care	19.2	1-69

### Survey Results at Time 1 and Time 2

#### Survey Responses

Table 3 shows mean scores for the survey scales, along with the scales’ internal consistencies. Cronbach alpha values ranged from .89 to .95, indicating excellent internal consistency reliability [43,44]. There was a moderate extent of agreement with respect to utility of and satisfaction with the client health record and portal messaging. Scores for involvement in the care process and for the portal overall indicated agreement between “neutral” (score of 0) and “agree” (score of 1). Mean scores for portal messaging with service providers were neutral (–0.06) at time 1 and slightly higher (0.25) at time 2.

#### Mean Survey Item Responses

Table 4 shows a fair degree of overlap in items with the highest and lowest means at time 1 and time 2. The items with the highest means (greatest endorsement) concerned the accuracy and convenience of the client health record (section A), the usefulness, timeliness, and adequacy of portal messaging (B), a willingness to invest effort in the intervention process (C), and convenience in accessing care or services (D). There was also evidence of a strong intention to use the portal in the future (E). Items with the lowest means (least endorsement) concerned actual use of the client health record (although this was moderate; section A) and portal messaging (to a fairly small extent; B). There was little evidence that the portal led to feelings of greater involvement in the care process (C), improved ability to express concerns to providers or enhanced relationships with providers (D), or reduced number of in-person visits (E).

**Table 3:** Survey scale scores at time 1 and time 2.

Survey section	No. of items	Cronbach alpha (times 1 and 2 combined)	Time 1 (n=15)		Time 2 (n=11)	
			Mean^a^	(SD)	Mean^a^	(SD)
Utility/satisfaction: client health record^b^	6	.89	3.68	0.76	3.14	1.01
Utility/satisfaction: portal messaging^b^	6	.90	2.62	1.08	3.22	1.11
Involvement in the care process^c^	10	.91	0.69	0.77	0.77	0.52
Impact of portal messaging with service providers^c^	7	.95	–0.06	0.67	0.25	0.99
Portal overall^c^	9	.90	0.61	0.41	0.40	0.97

^a^Some data are missing (a score was calculated only if a respondent had data for ≥50% of the items in the scale).

^b^Scale choices were to a great extent=5, to a fairly great extent=4, to a moderate extent=3, to a fairly small extent=2, to a small extent or not at all=1.

^c^Scale choices were strongly agree=2, agree=1, neutral=0, disagree= –1, strongly disagree= –2.

**Table 4:** Survey items with the highest and lowest means at time 1 and time 2.

Survey section	Time 1 (n=15)^a^		Time 2 (n=11)^a^	
	Item(s) with the highest mean	Item(s) with the lowest mean	Item(s) with the highest mean	Item(s) with the lowest mean
A. Utility/satisfaction: client health record^b^ *To what extent did you*...	Feel the client health record was accurate (there were no mistakes)? (mean 4.07)	Access your/your child’s health record? (mean 2.93)	Find it easy to locate relevant information in the client health record? (mean 3.82)	Access your/your child’s health record? (mean 2.45)
B. Utility/satisfaction: portal messaging^b^ *To what extent did you...*	Feel this feature was useful to you? (mean 3.60)	Communicate with a member of the provider team using portal messaging? (mean 1.70)	Feel responses to your portal messages were timely? (mean 3.83) Feel responses to your portal messages were adequate? (mean 3.83)	Communicate with a member of the provider team using portal messaging? (mean 2.00)
C. Involvement in the care process^c^ *Based on my portal* *experience...*	I am willing to invest effort in the intervention process (mean 1.20)	I believe the intervention process (ie, treatment plan) will be effective (due to greater involvement in the care process) (mean 0.40)	I am willing to invest effort in the intervention process (mean 1.27)	I believe the intervention process (ie, treatment plan) will be effective (due to greater involvement in the care process) (mean 0.18)
D. Impact of portal messaging with service providers^c^ *Because of the portal*...	I feel that accessing care or services is more convenient for me (mean 0.67)	My ability to express concerns and/or provide comments to providers has improved (mean –0.36) I have built a more open and trusting relationship with health care providers (mean –0.36)	I feel that accessing care or services is more convenient for me (mean 0.64)	My communication with my provider has improved (mean 0.10) I have built a more open and trusting relationship with health care providers (mean 0.10) My sense of trust/rapport with the clinical provider team has increased (mean 0.10)
E. Portal overall^c^	I intend to use the portal in the future (mean 1.47)	The portal reduced the number of in-person visits I made (mean –0.27)	I intend to use the portal in the future (mean 1.00)	The portal reduced the number of in-person visits I made (mean –0.36)

^a^Some data are missing (a score was calculated only if a respondent had data for ≥50% of the items in the scale).

^b^Scale choices were to a great extent=5, to a fairly great extent=4, to a moderate extent=3, to a fairly small extent=2, to a small extent or not at all=1.

^c^Scale choices were strongly agree=2, agree=1, neutral=0, disagree= –1, strongly disagree= –2.

### Transcript Themes

#### Caregiver Themes

Three themes arose from our analysis of the caregiver transcripts, with the nature of these changing slightly at time 2 as caregivers acquired more experience with the portal. These themes were information benefits, recommendations to increase use and utility, and scope of adoption and future vision.

With respect to information benefits, at time 1, caregivers indicated the usefulness of the portal in providing easy access to their child’s medical history, reports, and appointments, and how this saved them time. At time 2, the benefits of access to the information itself received somewhat greater emphasis—participants expressed an appreciation for having more detailed information and knowing the technical language, as they felt they could then communicate on a more level playing field with providers (eg, “[if] we can use accurate language, language that they understand, we get a lot better dynamic where they’re going to listen to us”).

There were many recommendations to increase use and utility, including being able to message all clinicians, receiving speedier notification of messages waiting for them in the portal (caregivers had to go into connect2care to get their emails, and notifications about awaiting emails took time to arrive) and better information about how to do things on the portal (eg, send a message), and having clarification of the scope of confidentiality and portal access after discharge. The recommendations also included uniform implementation of portal features (some caregivers did not have access to features that others did) and greater comprehensiveness in what was available (eg, medication information, care plans, and particularly reports from all clinicians). These comments may reflect differences in whether a caregiver’s service providers were set up on e-messaging.

The theme of scope of adoption and future vision concerned caregivers’ ultimate hopes for the portal. They were aware that the portal was a “work in progress” and “in its infancy,” and shared their visions of the portal in the future. They commented on the utility of cross-organization EHRs, connection to the adult health care system, provision of information about available programs, and personalization on a broad scale (eg, ability to upload one’s own information to the portal and receive targeted information). They also commented on the need for organizationwide adoption, where portal use was embedded in routine practice (eg, “it’s early days...but if just everybody could get on board...the opportunities for saving everybody’s time and aggravation in messaging are huge”).

#### Service Provider Themes

Four themes emerged from our analysis of the service provider transcripts. There were no differences in these themes at time 1 and time 2. First, the theme utility for families indicated that service providers saw the utility of the portal in setting up appointments and providing secure messaging. They were less sure about the impact of the portal on client engagement but felt that the portal provided a positive, inviting message to families about being engaged (eg, “messages of we want you to be engaged, we’re trying to reach out to you, we’re trying to have multiple ways of engaging with you”).

Second, service providers identified technical shortcomings in several areas, including lack of notification of emails (requiring repeated logons to check whether emails from caregivers had been received), and lack of ability to post vacation messages and upload attachments. Problems in formatting occurred when reports from the clinical system were uploaded to connect2care, and reports had to be changed in several places to ensure consistency in what clients saw.

The third theme dealt with uncertainties in portal use, related to lack of knowledge, comfort, or confidence in using the portal, in addition to some of the portal’s technical shortcomings. Service providers expressed uncertainty in knowing which families accessed the portal, whether reports were being used, whether a message was waiting or had been received, and what documents or materials families could access. They were also uncertain about their role in informing families about the portal and how to access it (eg, “it doesn’t seem like a big deal, but when I’m seeing 5, sometimes 6 people in a day, I don’t have that extra time to do that”).

The fourth service provider theme concerned use, effort, and investment in the portal. Due to low levels of perceived use by families, and the time, effort, and care required to see if messages were waiting and to produce user-friendly reports meeting professional standards, service providers expressed concerns about whether it was currently worth investing a great amount of time in the portal (eg, “it’s another thing to log in to,” “doesn’t make sense for me to go in every day, or multiple times a day, and check if in 6 months I’ve only ever got 2 messages”).

## Discussion

This study contributes to the growing literature on portals, as there has been no previous research, to our knowledge, on the use, utility, or impact of client portals in pediatric rehabilitation. Furthermore, studies of health care portals have largely focused on use and utility, rather than impact-related outcomes such as engagement in care or client-provider communication [27]. Compared with new portal annual adoption rates of 5% to 10% and access rates of 0.4 to 0.6 uses per month per user [45], the connect2care portal had an adoption rate of 12.4% and 2.5 logins a month per user over 9 months of exposure. There may be many reasons for the higher adoption and access rates, including the involvement of families in the development of the portal [2], effort put into informing families about the portal, and differences between acute care and pediatric rehabilitation contexts.

There was a moderate degree of perceived usefulness of and satisfaction with the EHR and e-messaging features, and evidence that the portal was perceived to provide useful access to the clinical record. Utility and satisfaction scores did not change much over time; however, there was only a 6- to 8-week time period between the 2 survey administrations, thus reducing the amount of new portal experience that was possible.

With respect to impact, there was some evidence that portal access facilitated caregivers’ perceptions of engagement in care, but this evidence was not strong (between neutral and agree, on average). As a point of comparison, van der Vaart et al [14] found that 44% of patients with rheumatoid arthritis felt more involved in their treatment as a result of portal access. Service providers in our study also indicated seeing little evidence of increased engagement. However, several caregivers indicated that reading available reports increased their understanding of the related technical language, and felt this enhanced their ability to engage in conversations about care with service providers.

We speculate that it takes time for portal use to have an effect on engagement in care (in this study, we collected time 1 engagement data after an average of approximately 9 months of portal exposure). At this early stage of portal use, caregivers appeared predominantly interested in the information they could access via the portal, although at the second measurement point 2 months later, there was some indication of greater appreciation of the actual content of the reports, which may reflect the fact that more and more clinical documentation was being added through the study period. With respect to impact on caregiver-provider communication, there was some evidence at time 2 of improved communication due to the introduction of e-messaging. We speculate that e-messaging had not been introduced long enough or widely enough to affect client engagement. Alternatively, caregivers may be already quite engaged in their child’s care, so that the portal may not make such a big difference in engagement compared with nonpediatric portals.

The qualitative themes were informative, as they allowed comparison of caregivers’ and service providers’ perspectives. There was a common emphasis on the utility of the information provided via the portal. As well, both groups questioned the extent of the impact on engagement in care at this early stage of portal rollout (corroborating survey data), and both groups recommended increasing portal use and utility by addressing technical shortcomings. These recommendations were related to the portal not meeting expectations for technology and not being as user friendly as desired.

The major difference between caregivers and providers was that caregivers focused on the scope of adoption of the portal system in the organization and expressed their hopes for the future of the portal with respect to their family’s life journey (the scope of adoption and future vision theme), whereas service providers were concerned about how to best manage their investment of time and effort (the use, effort, and investment in the portal theme). Both groups expressed a desire for the other group to use the portal to a greater extent: caregivers wanted to see organizationwide adoption, whereas some providers in our small sample questioned whether their investment in the portal was justified given low levels of perceived use. As well as full organizationwide adoption of the portal system, caregivers’ hopes for the future included greater personalization and comprehensiveness of the provided information. As recognized by caregivers, portals are in their infancy and the maturity of portals does not appear to be where it needs to be to improve quality of care and involve the patient in care decisions [27].

Planned future enhancements to the connect2care portal will address some of the hopes of caregivers, including the ability to import reports from other service providers, link to records from other hospitals, and receive materials and resources specifically targeted to their care needs [2].

### Study Strengths and Limitations

Strengths of this investigation include prospective data collection of login information starting at the portal’s introduction, the breadth of information collected using multiple methods, inclusion of both caregiver and provider perspectives, and focused examination of caregiver engagement and caregiver-provider communication using scales with excellent internal consistency. Study limitations include its descriptive nature, the relatively short time period between measurement points (6-8 weeks), and the relatively small number of respondents (18 caregivers and 5 providers). We likely did not have data saturation and therefore the robustness of the qualitative themes is uncertain [38,46], although triangulation with the survey data should be noted.

The study participants are likely representative of those who register for portal access but *not* of the Holland Bloorview client population overall, since people who register for portals are likely to be early adopters who embrace technology more readily. Research suggests that those who use patient portals are generally more highly educated, younger, more affluent, and have fewer medical problems than nonenrollees [28,47,48]. As with any innovation, expectations and technical difficulties play a role in adoption. Difficulties in getting portal technology to run as desired can affect portal use and may bias reports of utility, satisfaction, and impact [33,49], as may be the case in our study.

### Research and Organizational Implications

Future research directions include continued use of the survey to see whether there is evidence of increasing impact of the portal on client outcomes. Other directions include examination of expectations that are held about portal use and utility, and examination of the perceptions of young adult clients. For other researchers, this study has indicated the utility of analyzing login statistics and using a portal survey with demonstrated internal consistency. The study has also indicated the utility of an integrated knowledge translation approach, where clinicians, family members, and researchers come together to address an important applied question [50].

Organizationally, the findings indicate specific areas for improvements to the portal and its processes, many of which are being or have been addressed as the portal continues to be developed, including the establishment of processes to improve efficiency related to portal activities (such as reminder emails when a new message is received) and mechanisms to support the enrollment of all interested clients and caregivers. The findings also suggest the need for ongoing education about portal intents and for transparency in communication about implementation issues. The literature indicates that education is needed to manage expectations and enhance the extent of portal adoption [27]. Education and resources are needed to support providers in feeling confident that their clinical documentation is family-friendly and in using e-messaging effectively. Similarly, education is needed to enable caregivers to make the best possible use of the portal features.

Although caregivers in our sample saw the portal’s value, this was not yet the case for the service providers. This suggests that organizations may need to focus some of their efforts on ensuring that service providers see this value, as they are the ones who will share clinical notes and communicate via e-messaging—they too are partners in care. In directing attention to engaging clients or families, the engagement of service providers can be given less attention. As technology becomes more prominent in health care settings, it will be up to organizations to support uptake and demonstrate potential benefits for both parties. An important future direction at Holland Bloorview is therefore to share family stories with providers so that they can better understand the portal’s positive impact and feel that their time related to sharing information and communicating via the portal is worth the effort.

Lastly, the findings endorse the often-made statement that changing organizational culture takes time. Portal adoption is a process—not a one-time event—and requires a feedback loop (as provided by this study), allowing an organization to improve portal adoption through attention to the needs of the people who use it.

## Abbreviations

EHR: electronic health record
REDCap: Research Electronic Data Capture
